# Periodontal status, oral inflammatory biomarker, and relative expression of red complex bacteria in periodontitis-rheumatoid arthritis: a cross-sectional preliminary study

**DOI:** 10.3389/fcimb.2026.1824946

**Published:** 2026-06-01

**Authors:** Minessa Mahardika, Widya Rakhmawati, Benso Sulijaya, Sandra Olivia Kuswandani, Natalina Haerani, Suryo Anggoro Kusumo Wibowo, Yuniarti Soeroso, Sri Lelyati C. Masulili, Fatimah Maria Tadjoedin, Herlis Rahdewati, Cut Intan Safitri, Ines Augustina Sumbayak, Koichi Tabeta, Naoki Takahashi

**Affiliations:** 1 Periodontology Specialist Program, Department of Periodontology, Faculty of Dentistry, Universitas Indonesia, Jakarta, Indonesia; 2 Department of Periodontology, Faculty of Dentistry, Universitas Indonesia, Jakarta, Indonesia; 3 Basic, Translational, and Clinical Research in Periodontology Group. Faculty of Dentistry, Universitas Indonesia, Jakarta, Indonesia; 4 Graduate Research Programme, University College London (UCL) Eastman Dental Institute, London, United Kingdom; 5 Division of Rheumatology, Department of Internal Medicine, Faculty of Medicine, Universitas Indonesia - Dr. Cipto Mangunkusumo General Hospital, Jakarta, Indonesia; 6 Division of Periodontology, Department of Dentistry, Dr. Cipto Mangunkusumo General Hospital, Jakarta, Indonesia; 7 Division of Periodontology, Niigata University Graduate School of Medical and Dental Science, Niigata, Japan; 8 Division of Periodontology, Department of Oral Health Science, Faculty of Dental Medicine, Hokkaido University, Hokkaido, Japan

**Keywords:** dentistry, inflammatory biomarker, periodontal parameter, periodontitis, red complex bacteria, rheumatoid arthritis

## Abstract

**Objectives:**

To determine the differences in periodontal status—Probing Depth (PD), Clinical Attachment Loss (CAL), Bleeding on Probing (BOP); oral inflammatory biomarkers—Tumor Necrosis Factor Alpha (TNF-α), Interleukin 1 Beta (IL-1β), Prostaglandin E2 (PGE2); and the relative expression of red complex bacteria—*Porphyromonas gingivalis* (*Pg*), *Treponema denticola* (*Td*), and *Tannerella forsythia* (*Tf*) among groups with periodontitis and periodontitis accompanied by rheumatoid arthritis (RA).

**Materials and methods:**

A cross-sectional preliminary study by exploring periodontal clinical parameters, gingival crevicular fluid (GCF) inflammatory biomarker levels, and subgingival plaque relative expression of red complex bacteria. A total of 21 subjects, samples were taken one-time from patients with periodontitis (n=12) and periodontitis with RA (n=9). Biomarker levels were analyzed using the Enzyme-Linked Immunosorbent Assay (ELISA) and bacterial expression was measured using real-time polymerase chain reaction (RT-PCR). All periodontal parameters, GCF biomarkers, and bacterial expression were then analyzed accordingly.

**Results:**

No statistically significant differences (p > 0.05) were found for PD and CAL. BOP ​​was significantly higher in the periodontitis group than periodontitis+RA group. All biomarker levels were significantly increased in the periodontitis+RA group compared to periodontitis group. No significant difference in total bacterial expression (16sRNA) among groups. *Pg* and *Td* expression were significantly higher in the periodontitis group compared to periodontitis+RA group.

**Conclusions:**

In this exploratory study, patients with periodontitis and RA exhibited significantly higher inflammatory biomarkers levels, indicating an amplified inflammatory response. This exploratory study identifies numerical trends suggesting that systemic condition may be associated with a suppressed red complex profile in Indonesian RA patients.

## Introduction

Periodontitis is a chronic inflammatory disease that causes progressive periodontal tissue damage with a fairly high prevalence globally and also in Indonesia (Papapanou et al., 2018; Suratri et al., 2020; World Health Organization, 2022). This disease results from the interaction between pathogenic bacteria as etiological factors, host immune responses, and environmental influences as risk factors (Papapanou et al., 2018; Kwon et al., 2021; Santonocito et al., 2021; Darby, 2022). The World Health Organization (WHO), in the Global burden of severe periodontal disease in the Global oral health status report (2022), stated that the global prevalence of periodontitis is around 19% in humans aged over 15 years, starting in late adolescence, peaking at age 55 years and remaining high into old age (World Health Organization, 2022). Based on the 2023 Indonesian Health Survey (SKI), the prevalence of oral health problems for cases of easily bleeding gums in the age group ≥ 15 years reached 42.4% (Ministry of Health, Republic of Indonesia, 2023).

Several systemic diseases act as risk factors for periodontitis, and RA is thought to be one of them. Rheumatoid Arthritis is a chronic autoimmune disease that causes morbidity in the joints. If it affects the hand joints, it can lead to limitations in motor movement, specifically inhibiting the process of cleaning the oral cavity, which has the potential to worsen periodontal conditions (Aletaha et al., 2010). Periodontitis and RA have overlapping molecular inflammatory pathways. In both diseases, local tissue damage occurs involving the production of inflammatory mediators such as TNF-α, IL-1ß, and PGE2 (Ortiz et al., 2009; Beyer et al., 2018; Rahajoe et al., 2019; Bartold and Lopez-Oliva, 2020; Lehenaff et al., 2021; Nik-Azis et al., 2021; Chen et al., 2022). Rheumatoid arthritis (RA) and periodontitis share several pathological features, including bone and soft tissue destruction, as well as elevated levels of circulating inflammatory proteins (World Health Organization, 2022). Numerous studies and systematic reviews have explored the bidirectional relationship between periodontitis and RA, from both the microbial perspective as a causative agent and the inflammatory response as a host reaction. Periodontitis in RA patients has been reported, with a prevalence of two out of three patients experiencing moderate to severe forms of the disease. Severe periodontitis symptoms in RA patients have been significantly associated with increased levels of Anti-Citrullinated Protein Antibody (ACPA), alterations in the subgingival bacterial profile, and elevated levels of both systemic and oral inflammatory mediators (Gamel et al., 2017; Rahajoe et al., 2019; Hidayat et al., 2021).

One study on the relationship between periodontitis and RA in Indonesia showed a relationship between the prevalence and severity of periodontitis in RA patients based on clinical parameters (Susanto et al., 2013; Balci Yuce et al., 2017). There is no study in Indonesia that has explored the characteristic of RA and periodontitis at the molecular level, especially inflammatory biomarkers in GCF. This study provides an overview of the TNF-α, IL-1ß, and PGE2 levels in GCF, as well as CAL and BOP values ​​in patients with periodontitis with RA.

## Materials and methods

### Study design

The study was approved by the ethics committee for dental research of the Faculty of Dentistry Universitas Indonesia (No. 19/Ethical Approval/FKGUI/III/2024) and the ethics committee for health research, Dr. Cipto Mangunkusumo General Hospital - the Faculty of Medicine Universitas Indonesia (No. KET-758/UN2FI/ETIK/PPM.00.02/2024). The cross-sectional preliminary study was performed at the Periodontology Clinic, Dental Teaching Hospital Faculty of Dentistry, Universitas Indonesia and the Rheumatology Clinic, Dr. Cipto Mangunkusumo General Hospital. All participants were informed of the aims and methods of the study, and written informed consent was obtained in advance. In total, the study population included 21 participants: 12 systemically healthy periodontitis patients and 9 periodontitis with RA. The inclusion criteria for all groups were age of 35–59 years and diagnosis of periodontitis with interdental CAL detectable at ≥ 2 non‐adjacent teeth or buccal CAL ≥ 3mm with pocketing ≥ 3mm detectable at ≥ 2 teeth, but the observed CAL cannot be described to non‐periodontitis‐related causes. For the RA groups, a newly-confirmed diagnosis of RA (naïve, never had a therapy) done by a rheumatologist according to the 2010 ACR/EULAR classification. Data of systemic CRP and Disease Activity Score-28 (DAS-28) was collected in RA patients. The exclusion criteria for all groups were diabetes mellitus, hypertension, endocarditis, other autoimmune disease, HIV/AIDS, hepatitis, tuberculosis, pregnancy, smoking, use of antibiotic drugs and/or periodontal treatment within the last 3 months, and inability to perform oral hygiene.

All participants underwent a full-mouth periodontal examination by calibrated periodontists (M.M. and W.R.), assessing PD, CAL, and BOP. The PD and CAL measurements were performed with a UNC-15 periodontal probe (Hu-Friedy Co., Chicago, IL, USA) at 6 sites per tooth (mesiobuccal, mid buccal, distobuccal, mesiolingual, midlingual, and distolingual) for all teeth except the third molar, and the deepest one was recorded. The BOP was recorded as present or absent within 30 seconds of probing at 6 sites per tooth for all teeth.

### GCF sampling

GCF for sampling was taken at the deepest PD site. The site was isolated with cotton rolls to avoid saliva contamination. A paper point was inserted into the pocket for 30 seconds. Paper with blood contamination was discarded. Subsequently, the paper point was removed and immediately frozen at -80°C in an Eppendorf tube.

### Subgingival biofilm sampling

The sampling area was isolated with a cotton roll, supragingival plaque was eliminated using an excavator, scraped gently at the deepest PD site, and then placed on a paper point no. 15. Then it was put into an Eppendorf tube containing Phosphate Buffer Saline solution and labelled.

### Measurement of TNF-α, IL-1ß, and PGE2 using ELISA

GCF samples for TNF-α, IL-1ß, and PGE2 (Human interleukin-1β, ELISA Kit, Bioenzy, Indonesia) were assessed using enzyme linked immunosorbent assay (ELISA). The concentrations of TNF-α, IL-1ß, and PGE2 in the samples were determined using a standard curve. To enhance the transparency of the methodology, testing was conducted under optical density (OD) of 450nm in accordance with standard procedures with a detailed manufacturer’s protocol. Similar to data collection, instrument calibration, sample handling and storage methods, and analysis techniques adhered to the specified qualifications.

### Measurement of *Pg*, *Td*, and *Tf* using real-time PCR

Bacterial DNA was isolated from the subgingival plaque samples by following the manufacturer’s recommendations for the GENEzol reagent (General, Ltd, New Taipei City, Taiwan). In order to minimize potential errors in real-time polymerase chain reaction (PCR) testing, a precautionary measure was taken by duplicating samples. Subsequently, DNA synthesis was performed using a reverse transcription kit from Applied Biosystems.

The specific primer sequences utilized in this study, as shown for *Pg Forward primer: 5′-AGG CAG CTT GCC ATA CTG CG-3′, Reverse primer: 5′-ACT GTT AGC AAC TAC CGA TGT-3′*; *Td Forward primer: 5′-TAA TAC CGT ATG TGC TCA TTT ACA T-3′, Reverse primer: 5′-TCA AAG AAG CAT TCC CTC TTC TTC TTA-3′*; *Tf Forward primer: GCG TAT GTA ACC TGC CCG CA, Reverse primer: TGC TTC AGT GTC AGT TAT ACC T;* and total bacteria Forward primer: 5’TTAAACTCAAAGGAA TTGACGG3’, Reverse primer: 5’CTC ACG ACA CGA GCT GAC GAC 3’. This observation holds significance as it aids in assessing the specificity and successful amplification of a particular genetic target. The obtained data were subjected to analysis employing the 2^−ΔΔCT^ method.

### Statistical analysis

Due to the exploratory and pilot nature of this study, a formal power calculation was not performed. Statistical analysis was performed using the statistical software GraphPad Prism (version 10.4.1). Metric data were examined according to their normal distribution using the Shapiro-Wilk test. The comparison of normally distributed data was performed using an independent T-test, and non-normally distributed data were compared using the Mann-Whitney U test. A p-value of < 0.05 was considered statistically significant.

## Results

**Figure 1:**
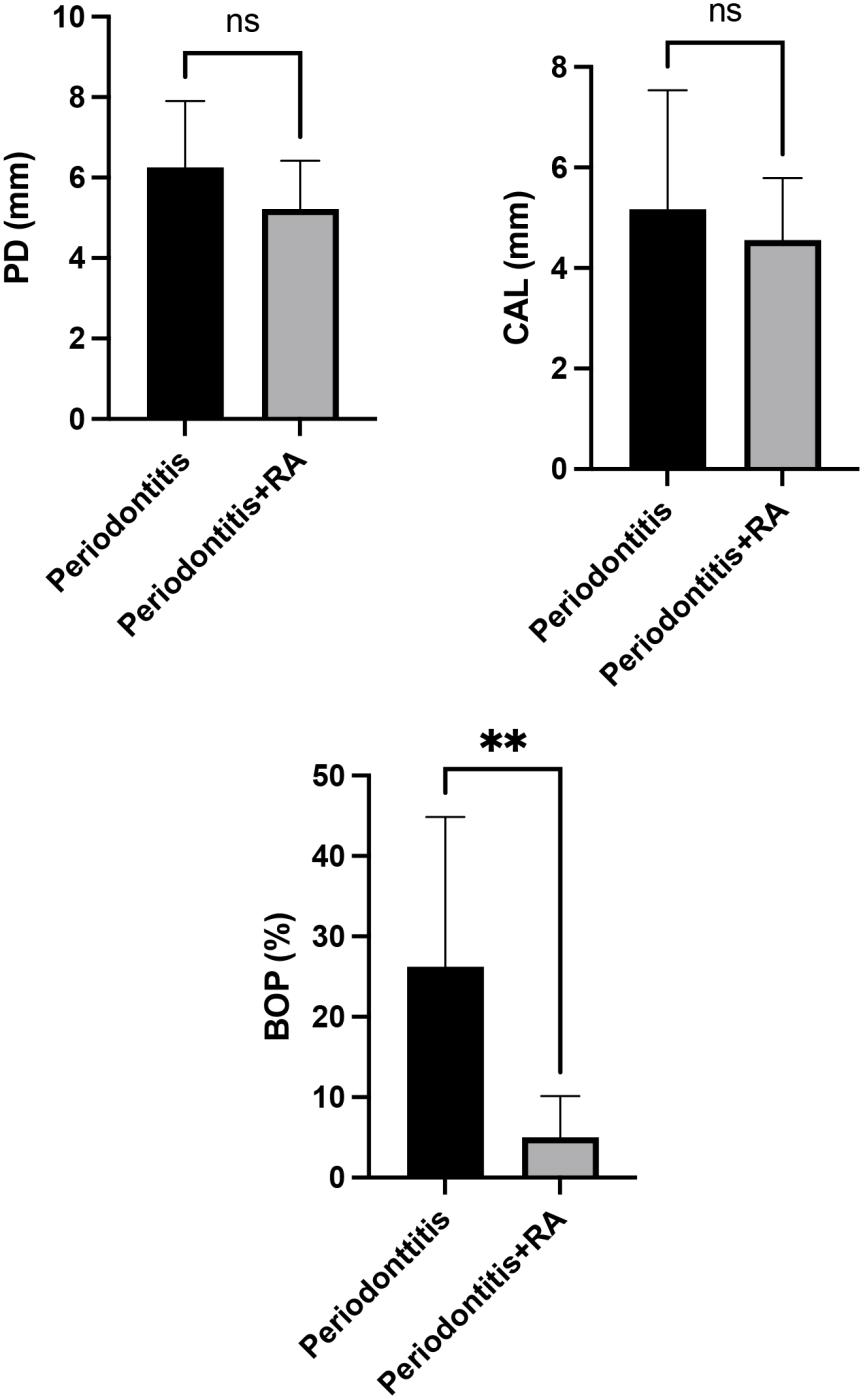
Periodontal clinical parameters. Mann-Whitney U test. N: 9–12 subjects per group. * and **, *P <*0.05; ns, non-significant.

**Figure 2:**
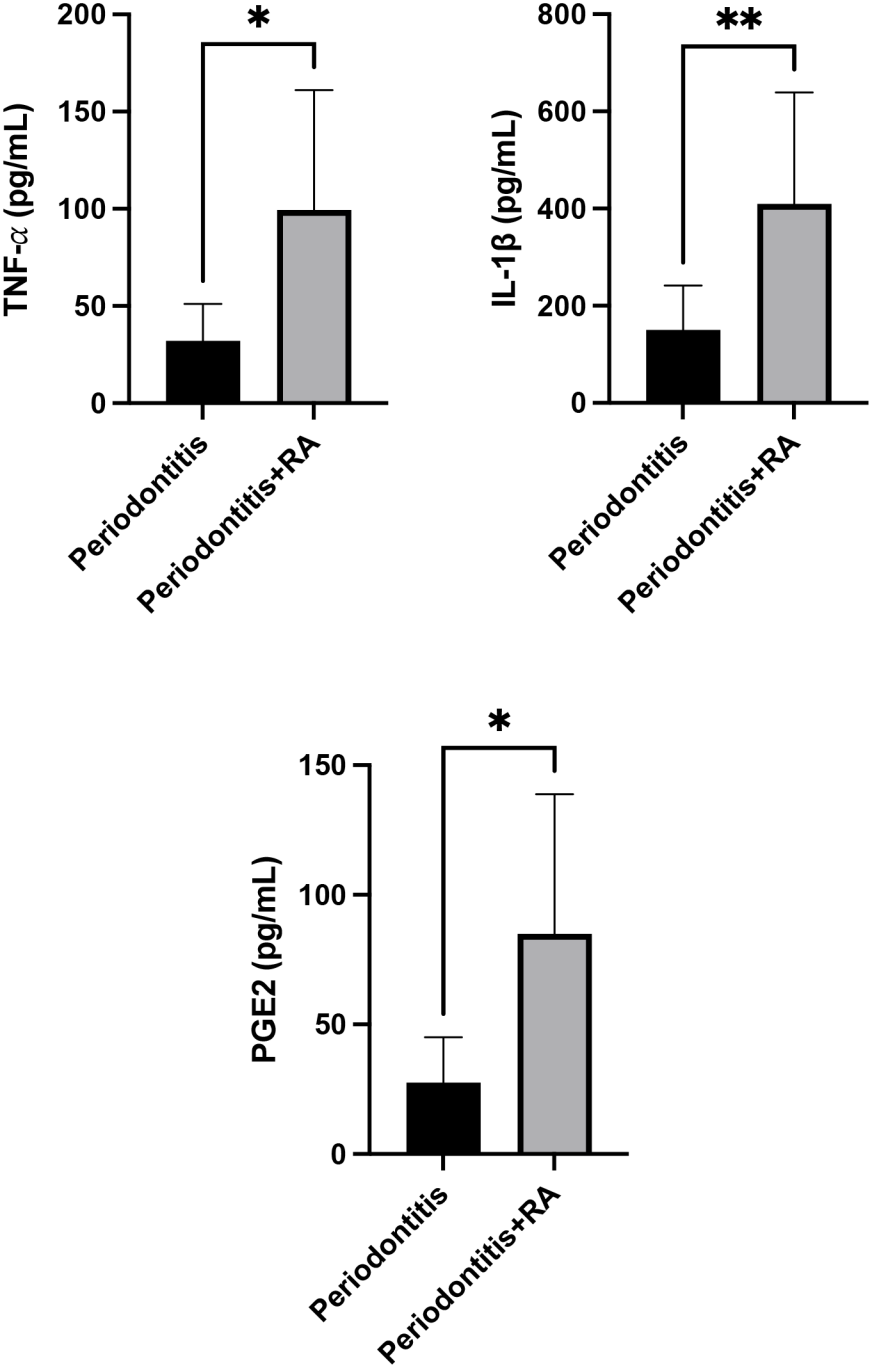
GCF inflammatory biomarkers. Mann-Whitney U test. N: 9–12 subjects per group. * and **, *P <*0.05; ns, non-significant.

Demographic data, including patient gender and age, are summarized in **Table 1**. A total of 21 participants were enrolled in this cross-sectional study and categorized into twelve patients periodontitis (P) (2 men and 10 women; mean age 48.17 ± 6.32; range 41–59 years), nine periodontitis with RA group (P + RA) (all women; mean age 42.44 ± 8.23; range 35–59 years). All participants demonstrated clinical signs of moderate-to-severe periodontal destruction, as their median of CAL was 4–5 mm. As summarized in **Tables 2** and **3**, clinical periodontal parameters and systemic inflammatory markers exhibited distinct numerical trends across the study cohorts. Both PD and CAL from the periodontitis group (6.25 ± 1.66mm; 5.17 ± 2.37mm) and periodontitis+RA (5.22 ± 1.20mm; 4.56 ± 1.24mm) showed no significant difference(p>0.05) (**Figure 1**, **Table 2**). Only BOP was found to be higher in periodontitis compared to periodontitis+RA group (**Figure 1**, **Table 2**). A statistically significant differences found only in TNF-α (p=0.005), IL-1β (p= 0.008), and PGE2 (p=0.013) levels between the periodontitis group and the periodontitis+RA group (**Figure 2**, **Table 3**).

**Table 1:** Demographic characteristics.

Characteristic	P (n =12) mean ± SD	P+RA (n =9) mean ± SD
Age (years)	48.17 ± 6.32	42.44 ± 8.23
Sex		
Female	10	9
Male	2	–
Systemic CRP (mg/L) DAS-28	- -	10.72 ± 9.54 4.59 ± 0.58

P, periodontitis; P+RA, periodontitis + rheumatoid arthritis; n, number of subjects; SD, standard deviation; CRP, C-reactive protein; DAS-28, Disease Activity Score-28; (-), RA-specific markers (CRP and DAS-28) were only assessed in the P+RA cohort to characterize systemic disease activity.

**Table 2:** Periodontal clinical examination.

Clinical parameters	P (n =12) median (Min-Max)	P+RA (n =9) median (Min-Max)	P value
PD (mm)	6.00 (4.00-9.00)	5.00 (4.00-8.00)	ns
CAL (mm)	5.00 (2.00-11.00)	4.00 (3.00-6.00)	ns
BOP (%)	15.30 (8.00-58.00)	3.80 (0.00-12.00)	0.001**

Mann-Whitney U test. * and **, significant p<0.05; ns, non-significant. P, periodontitis; P+RA, periodontitis + rheumatoid arthritis; n, number of subjects; Min, minimum; Max, maximum; PD, probing depth; CAL, clinical attachment loss; BOP, bleeding on probing.

**Table 3:** Inflammatory biomarker level.

Cytokine level	P (n =12) median (Min-Max)	P+RA (n =9) median (Min-Max)	P value
TNF->⍺ (pg/mL)	35.95 (10.03-57.15)	102.4 (24.47-231.5)	0.005*
IL-1β (pg/mL)	147.4 (49.96-267.7)	397.2 (100.5-869.7)	0.008**
PGE2 (pg/mL)	28.03 (7.959-54.49)	80.43 (18.53-191.7)	0.013**

Mann-Whitney U test. * and **, significant p<0.05. P, periodontitis; P+RA, periodontitis + rheumatoid arthritis; n, number of subjects; Min, minimum; Max, maximum; TNF->⍺, tumour necrosis factor alpha; IL-1β, interleukin 1 beta; PGE2, prostaglandin E2.

**Figure 3:**
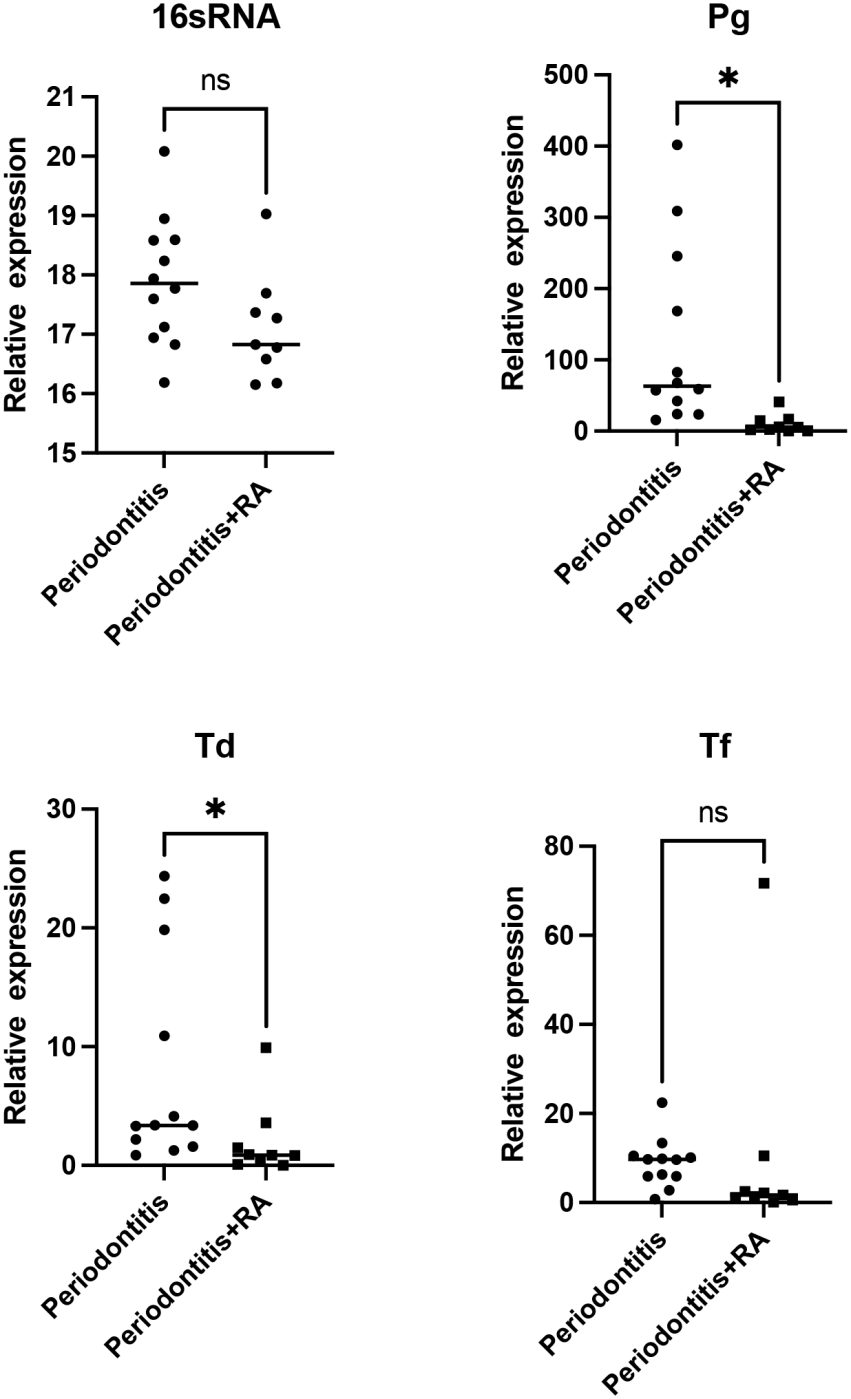
Relative expression of total bacteria (16sRNA) and red complex bacteria. Mann-Whitney U test. N: 9–12 subjects per group. *, *P <*0.05; ns, non-significant.

The relative expression of total bacteria and red complex bacteria values are presented in **Figure 3** and **Table 4**. The relative expression of total bacteria in each sample group showed no significant difference. The significant differences were found only in *Pg* (p=0.012) and *Td* (p=0.031) between the periodontitis group and the periodontitis+RA group. Overall, the microbial findings suggest a successional shift toward a less pathogenic subgingival profile (*Pg* and *Td*) in the periodontitis+RA group, though larger samples are required to confirm statistical correlations (**Figure 3**). **Table 1** shows the systemic serum C-Reactive Protein (CRP) levels was 10.72 ± 9.54mg/L and DAS-28 was 4.59 ± 0.58 in the periodontitis+RA group. It provides a biochemical baseline for the systemic inflammatory burden and moderate disease activity within this exploratory study.Discussion.

**Table 4:** Relative expression of total bacteria and red complex bacteria.

Relative expression	P (n =12) median (Min-Max)	P+RA (n =9) median (Min-Max)	P value
16sRNA	17.86 (16.19-20.08)	16.83 (16.15-19.03)	ns
*Pg*	63.21 (15.57-402.0)	5.79 (0.014-40.98)	0.012*
*Td*	3.37 (0.85-24.36)	0.85 (0.006-9.91)	0.031*
*Tf*	9.67 (0.76-22.45)	1.71 (0.10-71.68)	ns

Mann-Whitney U test. *, significant p<0.05; ns, non-significant. P, periodontitis; P+RA, periodontitis + rheumatoid arthritis; n, number of subjects; Min, minimum; Max, maximum; Pg, Porphyromonas gingivalis; Td, Treponema denticola; Tf, Tannerella forsythia.

The 2023 National Health Survey report showed a higher prevalence of oral health problems, bleeding gums, in women (7.3%) compared to men (6.4%) in Indonesia (Ministry of Health, Republic of Indonesia, 2023). Pavlov-Dolijanovic et al. (2023) in their review explained that the ratio of women to men in Young and Old onset Rheumatoid Arthritis (YORA) was 3:1 (Pavlov-Dolijanovic et al., 2023). Periodontal clinical parameters in this study included the PD, CAL, and BOP. The present study showed that no significant difference in CAL values between periodontitis and periodontitis+RA group. Zhao et al. (2018) in their systematic review state that periodontitis significantly increases the severity of RA, but RA itself does not significantly affect the development of periodontitis (Bunte and Beikler, 2019). Research by Susanto et al. (2013) showed no significant differences in most periodontitis severity parameters between RA patients and controls (Susanto et al., 2013). Studies from Jung et al. (2018) show that RA patients showed significant improvement in periodontal parameters after a non-surgical periodontal therapy is performed (Xiao et al., 2021). In this study, the BOP value of periodontitis patients was significantly higher than that of patients with periodontitis+RA group (**Figure 1**, **Table 2**). This finding suggests that similar periodontal condition (PD and CAL) might show different state of clinical inflammation, especially in patient with systemic condition.

This study showed comparative analysis results that were significantly different in each inflammatory biomarker level between periodontitis patients and periodontitis+RA, where the lowest median value was mostly seen in periodontitis patients. TNF-α, IL-1β, and PGE2 levels were lower in periodontitis patients as compared to periodontitis+RA group. This finding aligns that local and systemic inflammation can increase the inflammatory effect by RA conditions in periodontitis patients (**Figures 1**–**3**; **Tables 1**-**4**). The hypothesis proposed by Golub et al. in 2006 stated that in individuals who initially experience a local chronic inflammation in the form of periodontitis, which produces anti-cyclic citrullinated peptide antibodies, when joint inflammation occurs later, which produces citrullination, the subsequent antibody response can be very strong (Bartold and Lopez-Oliva, 2020). The results of this study are in accordance with this hypothesis, where the levels of TNF-α, IL-1β, and PGE2 in GCF are higher in periodontitis+RA patients. Research from Xiao et al. (2021), Arvikar et al. (2021), Cosgarea et al. (2019), and a literature review by Bartold and Lopez-Olivia (2020) stated that there were high levels of TNF-α, IL-1β in patients with periodontitis with RA compared to patients with periodontitis without RA (Kurgan et al., 2016; Balci Yuce et al., 2017; Özçaka et al., 2018; Cosgarea et al., 2019; Bartold and Lopez-Oliva, 2020; Arvikar et al., 2021; Xiao et al., 2021). A study by Kurgan et al. (2016) showed statistically higher PGE2 levels in the periodontitis group with RA compared to the periodontitis group without RA in their research results (Kurgan et al., 2016).

Several reports from previous studies have shown improvements in RA parameters and disease activity after non-surgical periodontal treatment, thus indicating the need for periodontal treatment as additional treatment for RA patients with periodontitis (Ortiz et al., 2009; Bartold and Lopez-Oliva, 2020; Bezerra et al., 2022). This is interesting because host-modulating treatments are also additional treatments for periodontitis (Chondros et al., 2009). However, this present study was an exploratory study on periodontitis patient and periodontitis with RA naïve patients. Nor treatment or intervention was given in this study. Chronic inflammation can also have the effect of suppressing the host defence system, which makes periodontal infection worse because the host loses its defence capacity (Persson and Persson, 2005).

The comparative analysis of total bacteria (16sRNA) in each sample group showed no significant difference. This can be related to the high differences in bacterial composition, with the dominant types of bacteria, whether pathogenic, commensal, or opportunistic, varying between healthy individuals, those with periodontitis, and those with systemic conditions such as rheumatoid arthritis (Johansson et al., 2016; Bhardwaj, 2018; Bhatsange and Rajput, 2024). The relative expression of red complex bacteria (*Pg, Td, Tf*) shown in **Figure 3** was significantly higher, especially for *Pg* and *Td* bacteria in periodontitis+RA as compared to periodontitis group. This tendency may be due the antibody reaction against *Pg*, resulting in a decrease in *Pg* in the periodontitis+RA samples. A study conducted by Johansson et al. determine whether antibodies against *Pg* were presented before the onset of RA. *Pg* bacteria are suspected to play a role in triggering RA by inducing the production of anti-citrullinated protein antibodies (ACPA), which are the main markers of this disease. ACPA antibodies are known to appear several years before RA symptoms manifest, indicating that an immune system imbalance occurs long before RA symptoms develop, with the likely initial site being mucosal tissues such as the gingiva (Quirke et al., 2014; Kussmann et al., 2021). Nevertheless, this study did not investigate the ACPA and the reason of lower *Pg* expression in periodontitis+RA group compared to periodontitis group remains unclear.

*Td* bacteria are often found alongside other periodontal pathogens in RA patients. The prevalence of *Td* and other periodontal pathogens may vary depending on the type of RA treatment (Kussmann et al., 2021). *Td* bacteria frequently interact with other periodontal pathogens such as *Pg* and *Tf*, which are collectively known as the red complex. *Td* expression was higher in periodontitis group compared to periodontitis+RA group (**Figure 3**, **Table 4**). We assumed that the microbial shift occurs as the local inflammation associates to systemic disease. The composition and microbial interactions may differ between patients with and without RA, potentially affecting the relative expression of *Td* detected (Inchingolo et al., 2023). In supporting the evidence of periodontitis and RA association (Kay-Arne et al., 2026), it must be emphasized that in this study, limited number of subject may be insufficient to detect stable or permanent microbiological successions. Consequently, the observed numerical reductions in Red Complex bacteria within the periodontitis+RA group might be interpreted as hypothesis-generating observations. In addition, variables such as overall RA disease duration was not controlled in this study that may have influenced the subgingival microbial profiles.

In this study, systemic inflammation was monitored via serum CRP levels and DAS-28. CRP serves as a reliable proxy for systemic inflammatory burden, while DAS-28 represents the disease activity score. Notably, CRP level (10.72 ± 9.54mg/L) and DAS-28 (4.59 ± 0.58) in the periodontitis+RA group shows biochemical baseline for the systemic inflammatory burden and moderate disease activity within this exploratory study. This biochemical data, paired with local periodontal markers like BOP and PD, suggests that the microbial alterations observed (e.g., shifts in *Pg* and *Td* abundance) are possibly linked to the combined influence of systemic condition and the host’s inflammatory state.

Several limitations should be considered when interpreting the findings of this preliminary study. As noted that the sample size is limited, we cannot rule out the influence of systemic factor, the observed microbial dysbiosis in the periodontitis+RA group aligns with the ‘successional shift’ patterns often seen in periodontal-rheumatoid transitions. Future prospective studies should incorporate other groups such as, periodontitis VS periodontitis+RA (active and remission), healthy perio with/without RA, also a longitudinal study design and drugs regime to further isolate the impact of multifactorial effect.

## Conclusions

In conclusion, except the BOP and inflammatory cytokines, this exploratory study did not find differences in all parameters between the periodontits and periodontitis+RA groups. This exploratory study identifies numerical trends suggesting that systemic condition may be associated with a suppressed red complex profile in Indonesian RA patients. These preliminary findings highlight the potential to influence the subgingival niche, though larger-scale studies are necessary to confirm these observations and establish definitive statistical correlations. Due to the small sample size and lack of statistical significance in several parameters, these findings cannot be generalized and should be interpreted as hypothesis-generating for future, adequately powered longitudinal studies in the Indonesian population.

## Data Availability

The raw data supporting the conclusions of this article will be made available by the authors, without undue reservation.
